# Multiple massive neurofibromas of lumbosacral plexus with intraspinal and pelvic extension

**DOI:** 10.11604/pamj.2014.17.67.3869

**Published:** 2014-01-27

**Authors:** Ali Akhaddar, Abad-Cherif El-Asri

**Affiliations:** 1 Department of Neurosurgery, Avicenne Military Hospital, Marrakech, Morocco; 2 University of Mohammed V Souissi, Rabat, Morocco

**Keywords:** Neurofibromas, lumbosacral plexus, sciatic pain, constipation

## Image in medicine

A 26-year-old man, previously healthy, presented with a 6-month history of bilateral sciatic pain and frequent constipation but no urinary symptoms. Physical examination revealed tenderness of the lumbosacral region with radiating pain along the sciatic nerve and the perinea without motor weakness of the inferior legs. No cutaneous lesions were noted especially no café-au-lait macules. Magnetic resonance imaging of the lumbosacral area showed multiple intracanalar lesions of the lumbosacral spine causing scalloping of posterior parts of all the sacral vertebrae and neural foraminal widening with bilateral and symmetric extension to the pelvic region. Biologic data were normal. Chest radiography followed by computed tomography revealed an axillary thoracic tumor (5 cm on diameter) which was biopsied and histologically identified as benign neurofibroma. Brain MRI showed no other cranial nerve tumors and there was no family history of neurocutaneous lesions. Because the patient had mild symptoms without neurological deficits, no surgery was performed for the sacral neurofibromas. Neurofibromas are one of the major characteristics of type 1 neurofibromatosis. They can develop from the Schwann cells or fibroblasts of any peripheral nerve. Massive intra-extraspinal cases characterized by dumbbell neurofibromas as in our case are rare and maybe asymptomatic for a long time. It is crucial to screen the entire spinal axis to detect all neurofibromas. Symptomatic cases justify surgical treatment but the decision for surgery and the surgical approach is quite complex because neurofibromas occupy a large spinal and extraspinal regions.

**Figure 1:**
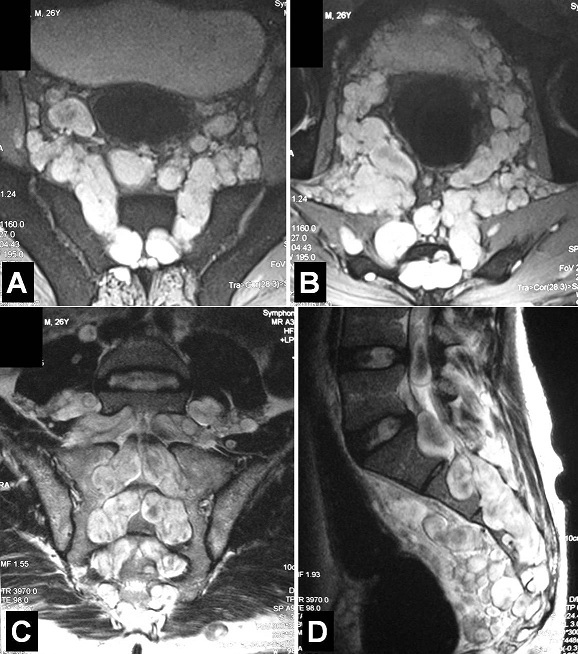
T2-weighted MRI of the lumbosacral area on axial (A and B), coronal (C) and sagittal (D) views. Multiple intracanalar hyperintense lesions of the lumbosacral spine causing scalloping of posterior parts of all the sacral vertebrae and neural foraminal widening with bilateral and symmetric extension (dumbbell formation) to the pelvic region

